# Large-scale, high-resolution electrophysiological imaging of field potentials in brain slices with microelectronic multielectrode arrays

**DOI:** 10.3389/fncir.2012.00080

**Published:** 2012-11-14

**Authors:** E. Ferrea, A. Maccione, L. Medrihan, T. Nieus, D. Ghezzi, P. Baldelli, F. Benfenati, L. Berdondini

**Affiliations:** ^1^Department of Neuroscience and Brain Technologies, Istituto Italiano di TecnologiaGenoa, Italy; ^2^Department of Experimental Medicine, Università di GenovaGenoa, Italy

**Keywords:** high-density electrode array, brain slices, functional imaging, local field potentials, epilepsy

## Abstract

Multielectrode arrays (MEAs) are extensively used for electrophysiological studies on brain slices, but the spatial resolution and field of recording of conventional arrays are limited by the low number of electrodes available. Here, we present a large-scale array recording simultaneously from 4096 electrodes used to study propagating spontaneous and evoked network activity in acute murine cortico-hippocampal brain slices at unprecedented spatial and temporal resolution. We demonstrate that multiple chemically induced epileptiform episodes in the mouse cortex and hippocampus can be classified according to their spatio-temporal dynamics. Additionally, the large-scale and high-density features of our recording system enable the topological localization and quantification of the effects of antiepileptic drugs in local neuronal microcircuits, based on the distinct field potential propagation patterns. This novel high-resolution approach paves the way to detailed electrophysiological studies in brain circuits spanning spatial scales from single neurons up to the entire slice network.

## Introduction

Electrophysiological recordings in brain slices are used in a wide range of studies to characterize neuronal circuits and signaling mechanisms or to investigate pathogenic states and rescue strategies in neuropharmacology. Currently available *in vitro* electrophysiological methods allow neuroscientists to record either the membrane potential of a single neuron or the extracellular field potentials generated by the superposition of local transmembrane currents flowing through neuronal compartments of multiple neurons (Nicholson and Freeman, 1975; Mitzdorf, 1985). However, these existing approaches have limitations in their ability to accurately study the spread of activity in larger brain circuits. To achieve large-scale high-resolution electrophysiological recordings it is imperative to increase the size of the recording area whilst, at the same time, the spatio-temporal resolution of these recordings remains high, ensuring high fidelity recording of the network dynamic under investigation (Chrobak and Buzsaki, 1998). Interestingly, low frequency extracellular signals reflect important global activity features in brain circuits that are still poorly understood. These local field potentials (LFPs) reflect the activity of several neurons and they can propagate between connected brain regions to influence the excitability of local networks (Logothetis et al., 2001; Buzsaki, 2010; Panzeri et al., 2010). Furthermore, characteristic LFP propagations are known to occur during epileptic seizures in cortico-hippocampal circuits (Barbarosie and Avoli, 1997). In this respect, acute cortico-hippocampal slices represent an established *in vitro* model for the study of neurophysiological processes underlying epileptogenesis (Cohen et al., 2002) and for screening potential anticonvulsant drugs (Hill et al., 2010; Gonzalez-Sulser et al., 2011). Conventional arrays of microelectrodes have been extensively used to record network spiking and LFP activity in brain slices (Shimono et al., 2000; Egert et al., 2002), but they are limited in array size and electrode density due to technical constraints in individually routing each electrode. Recently, novel electrode array technologies have opened new exciting opportunities to design significantly larger arrays that enable recordings at much higher resolution (Baker, 2010). These novel technologies are taking advantage of specifically designed microelectronic circuits that are either used in hybrid configurations for connecting large electrode arrays realized with conventional microfabrication methods, or for monolithic microelectronic chips integration embedding thousands of electrodes with the adapted read-out circuits. Based on the hybrid approach, a 512-channel array has been recently used to record at single cell resolution from the ganglion cell layer in the isolated primate retina (Field et al., 2010) and a flexible device with 360 electrodes was demonstrated to be effective in mapping cortical activity *in vivo* (Viventi et al., 2011). Using the monolithic microelectronic approach, a chip enabling to use 126 electrodes arbitrarily selected over an array of 11,011 microelectrodes has been used to record extracellular potentials from Purkinje cells in acute cerebellar slices (Frey et al., 2009) as well as to achieve sub-cellular resolution recordings in neuronal cultures (Heer et al., 2007). As an alternative to arrays of metallic electrodes, high-resolution field-effect-transistor (FET) arrays have also been developed, potentially offering even higher electrode integration densities (Hutzler et al., 2006). However, so far none of these multielectrode array (MEA) designs provide large enough recordings areas in order to achieve high enough spatial and temporal resolution to characterize fine-grain properties of neural activity in large brain circuits.

**Figure 1:**
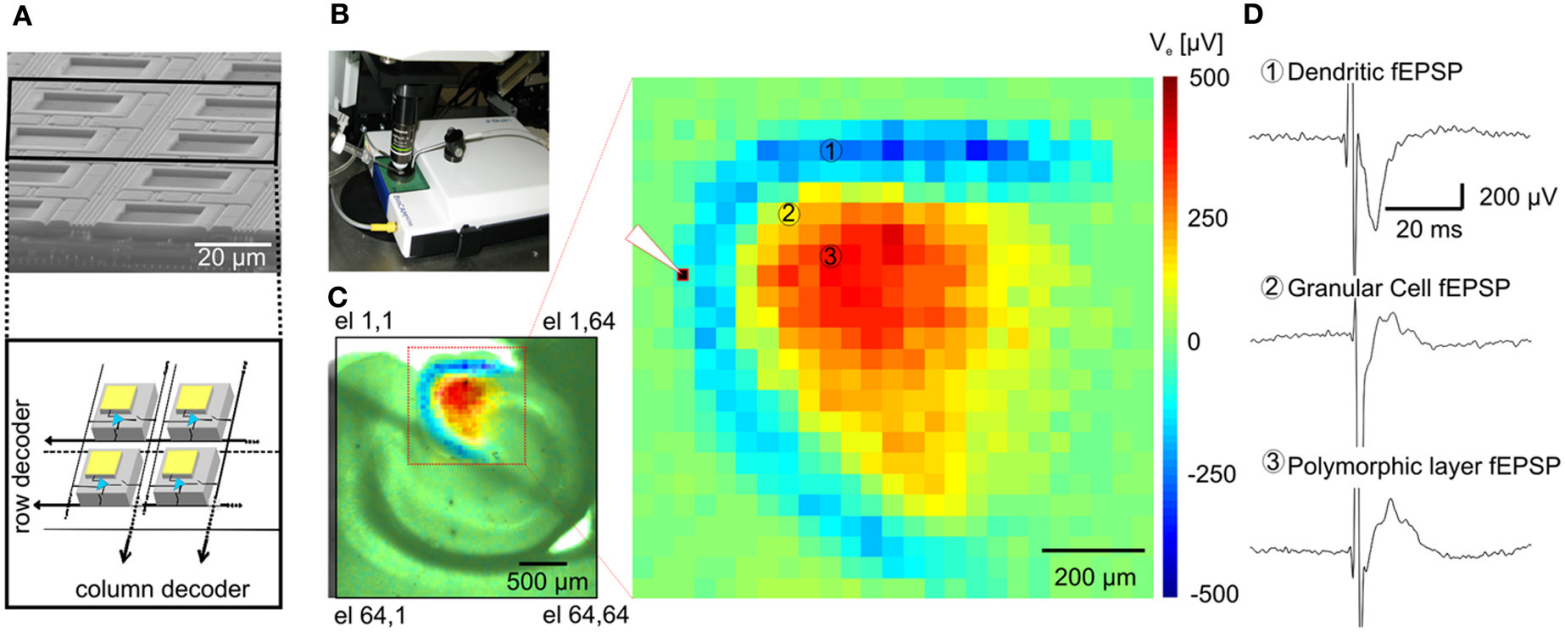
**Functional imaging of the dentate gyrus. (A)** On the top, Scanning Electron Microscopy (SEM) picture of a cross-section of an area of the APS-MEA chip; on the bottom, schematics of the amplifying and multiplexing circuitries integrated below each electrode. **(B)** APS-MEA system coupled with an upright microscope for field electrode stimulation and simultaneous VSD recordings. **(C)** A cortico-hippocampal slice over the active area of the chip with superimposed color-coded fEPSP activity and close-up on the activated area (the white tip indicates the site of stimulation). **(D)** Electrophysiological traces of fEPSPs recorded by three APS-MEA electrodes located in the dendritic layer of the dentate gyrus (electrode 1), in the granular cell layer (electrode 2) and in the polymorphic layer (electrode 3).

Here, we demonstrate for the first time the feasibility of large-scale electrical recordings of extracellular field potentials in brain slices using a large and dense array consisting of 4096 microelectrodes. This system enables us to perform functional electrophysiological imaging at unprecedented resolution to visualize and quantify both spontaneous or evoked spiking activity and LFPs from cortico-hippocampal slices. Our approach is based on an active-pixel sensor multielectrode array system (APS-MEA chip, Figure 1A) that records simultaneously from 4096 electrodes at a sampling rate of 7.7 kHz. The system provides 21 μm inter-electrode distance and 21 × 21 μm^2^ electrode size arranged in a squared area of 2.6 × 2.6 mm^2^ and it has been previously established for extracellular recordings of spiking activity from dissociated neuronal cultures (Berdondini et al., 2009). This platform can be coupled with conventional electrode wire recordings and fluorescence functional imaging for detailed electrophysiological studies in brain slices that take advantage from the complementary recording resolution and sensing capabilities of these methods (Figure 1B).

## Results

### Validation of the large-scale recording capabilities on cortico-hippocampal slices

The large-scale active area of our high-resolution electrode array chip was used to record extracellular activity in transverse mouse brain slices encompassing the hippocampus (Figure 1C), perirhinal and entorhinal cortices.

**Figure 2:**
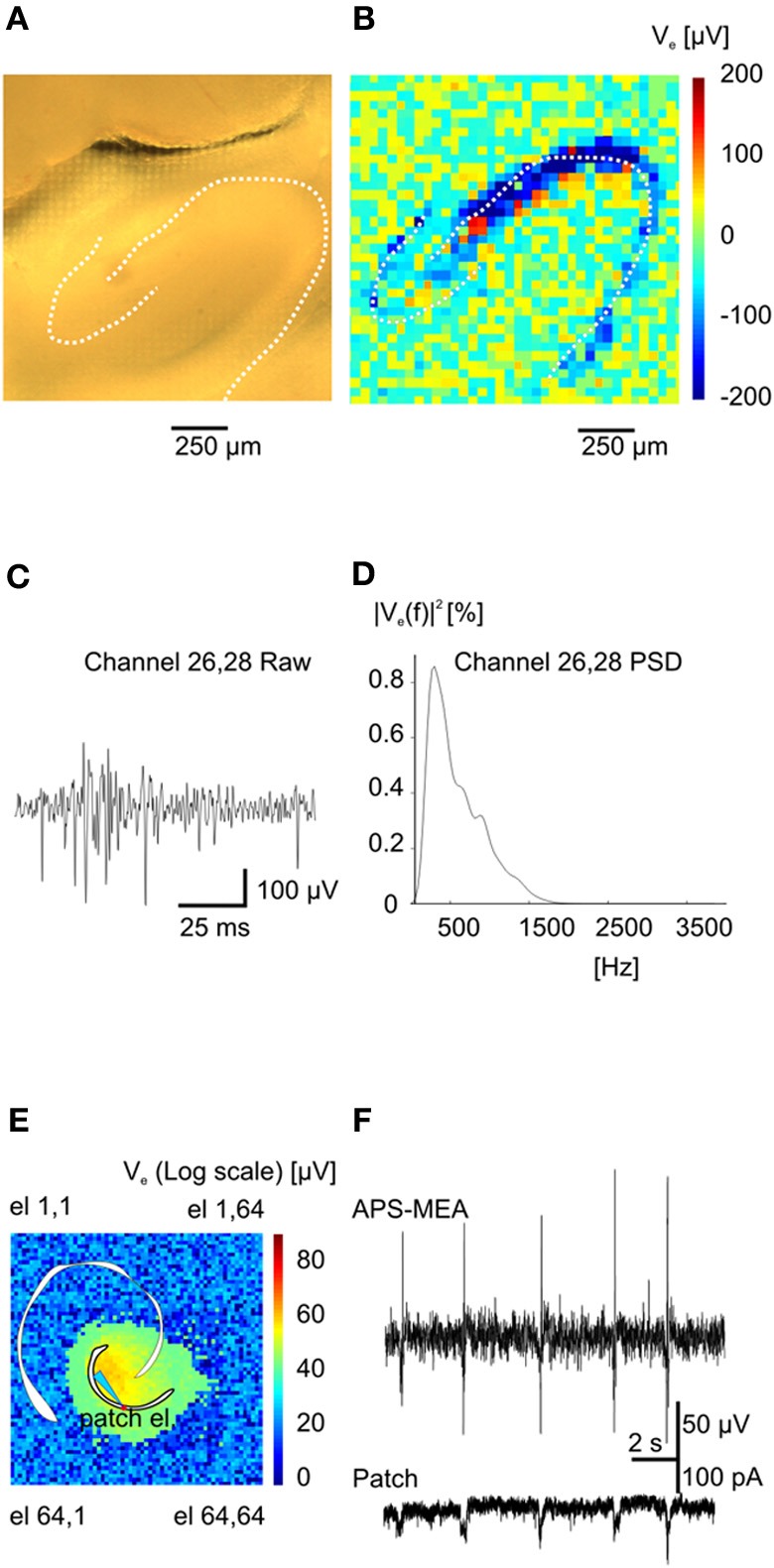
**Functional imaging of high frequency (action potentials) and low frequency events (Inter-Ictals). (A)** Hippocampal slice over the recording area. **(B)** Absolute maximum values of an action potential train (burst) at the various electrodes. **(C)** Burst event recorded on electrode 26–28. **(D)** Power spectral density for the same electrode. **(E)** Color-coded map of maximum activity of spontaneous epileptic-form events. The light-blue electrode is indicating the position of the patched cell whereas the red circle is indicating the correspondent pixel. **(F)** Simultaneous recording of extracellular activity with one pixel of the APS-MEA (top trace) and intracellular activity with a patch electrode (bottom trace).

Upon electrical stimulation of the perforant path, we recorded field excitatory postsynaptic potentials (fEPSPs) in the dentate gyrus (DG). Remarkably, the spatial distribution of the fEPSP recorded from the DG upon electrical stimulation of the perforant path perfectly matched the anatomical outline of the DG (Figure 1C). The local polarity of the fEPSP corresponds to current sinks and sources located in the dendritic, granule cell, and axonal layers respectively (Figure 1D). Moreover, both spiking and LFPs (Figures 2A–F) during spontaneous and evoked activities can be recorded, and viable brain slices were maintained for the entire duration of our experiments, up to 90 min. This viability was validated by performing parallel APS-MEA recordings and intracellular patch-clamp recordings from the tissue contacting the electrode array and neurons located near the upper side of the slice, respectively, and by synchronizing the entire slice activity with the help of the voltage-gated potassium blocker 4-aminopyridine (4AP) (Rutecki et al., 1987; Avoli et al., 1996; D'Antuono et al., 2002). The patch-clamp responses were both spatially and temporally tightly correlated with the fEPSP observed with the APS-MEA, thus demonstrating that our method yields accurate and reliable extracellular measures (Figures 2E,F).

**Figure 3:**
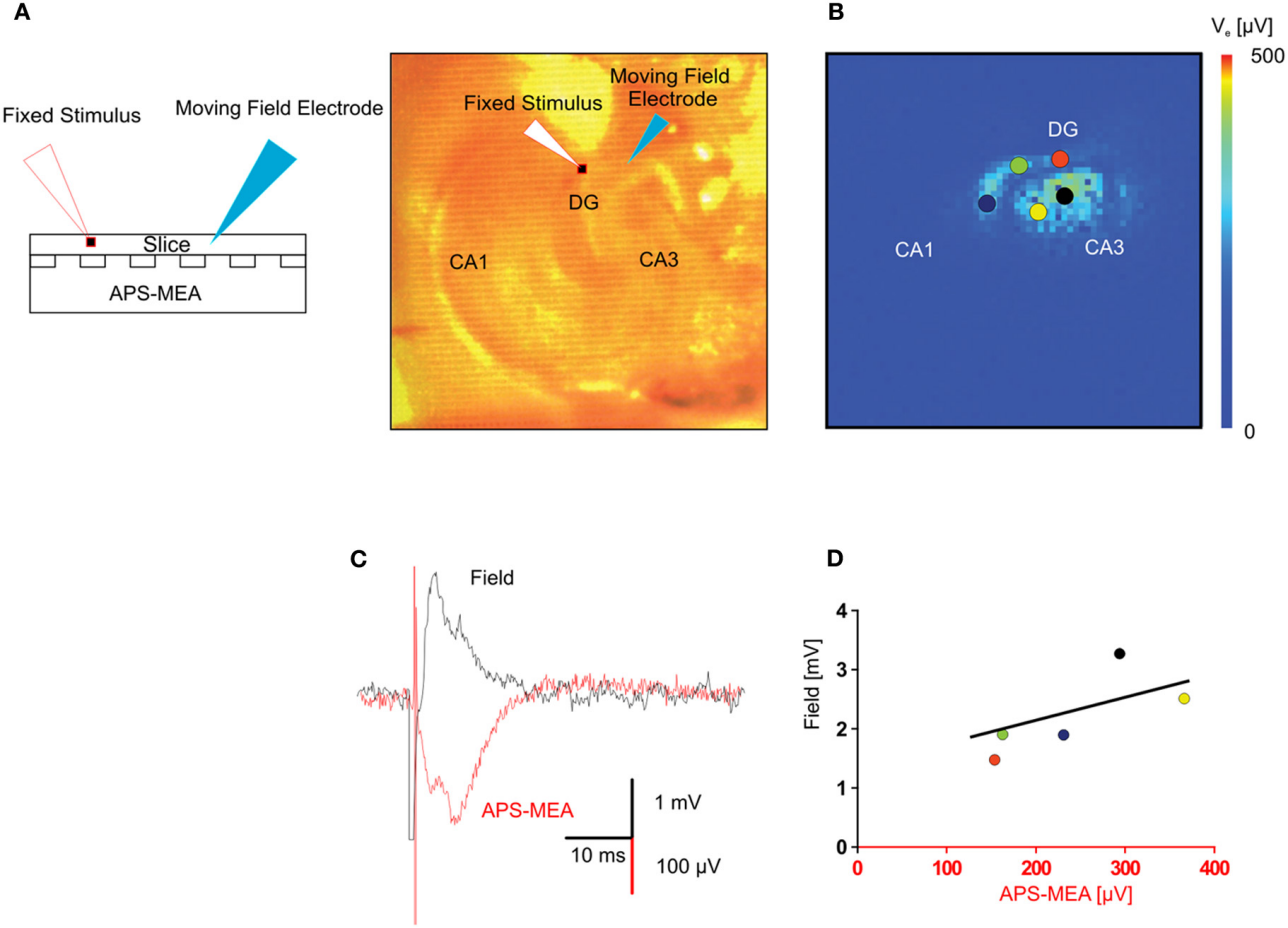
**Validation of the LFP recorded by means of high-density micro-electrode arrays through standard single field electrode recordings. (A)** The picture represents the adopted experimental configuration. The hippocampal slice is placed on the electrode array and two electrodes, one for extracellular stimulation the other for extracellular recording are micropositioned on the top of the slice. **(B)** Color-coded map of the maximal activation with superimposed circles showing the positions of the extracellular recording electrode. **(C)** In the same (x, y) position, the fEPSP recorded with one electrode of the APS-MEA had a signal to noise ratio similar to the fEPSP recorded with the conventional single extracellular electrode placed on top of the slice. The APS-MEA trace was inverted to be visually compared with the LFP recorded by a micropositioned extracellular electrode. Signals have slightly different shapes because they recorded at different heights in the brain slice (LFP at the top, APS-MEA at the bottom). **(D)** Linear fitting of the relationship between max amplitudes of the signal recorded with APS-MEA and with the conventional extracellular electrode in the same position.

To further validate the recording quality of our chip, we compared extracellular signals recorded using our microelectrodes with those obtained using classical field electrode positioned in the same slice. Voltage traces of electrically evoked responses recorded with a single wire electrode and with the corresponding electrode of the array, i.e., same (x, y) position, are shown in Figure 3A. The stimulating electrode was kept in the same position in the perforant path, whereas the recording single wire electrode was moved from the outer molecular layer to the hilus in order to correlate the evoked responses between five locations on the same plane (Figure 3B). This yielded responses with very similar shapes and signal-to-noise ratio (Figures 3C,D), indicating the high recording quality of our microelectrodes.

**Figure 4:**
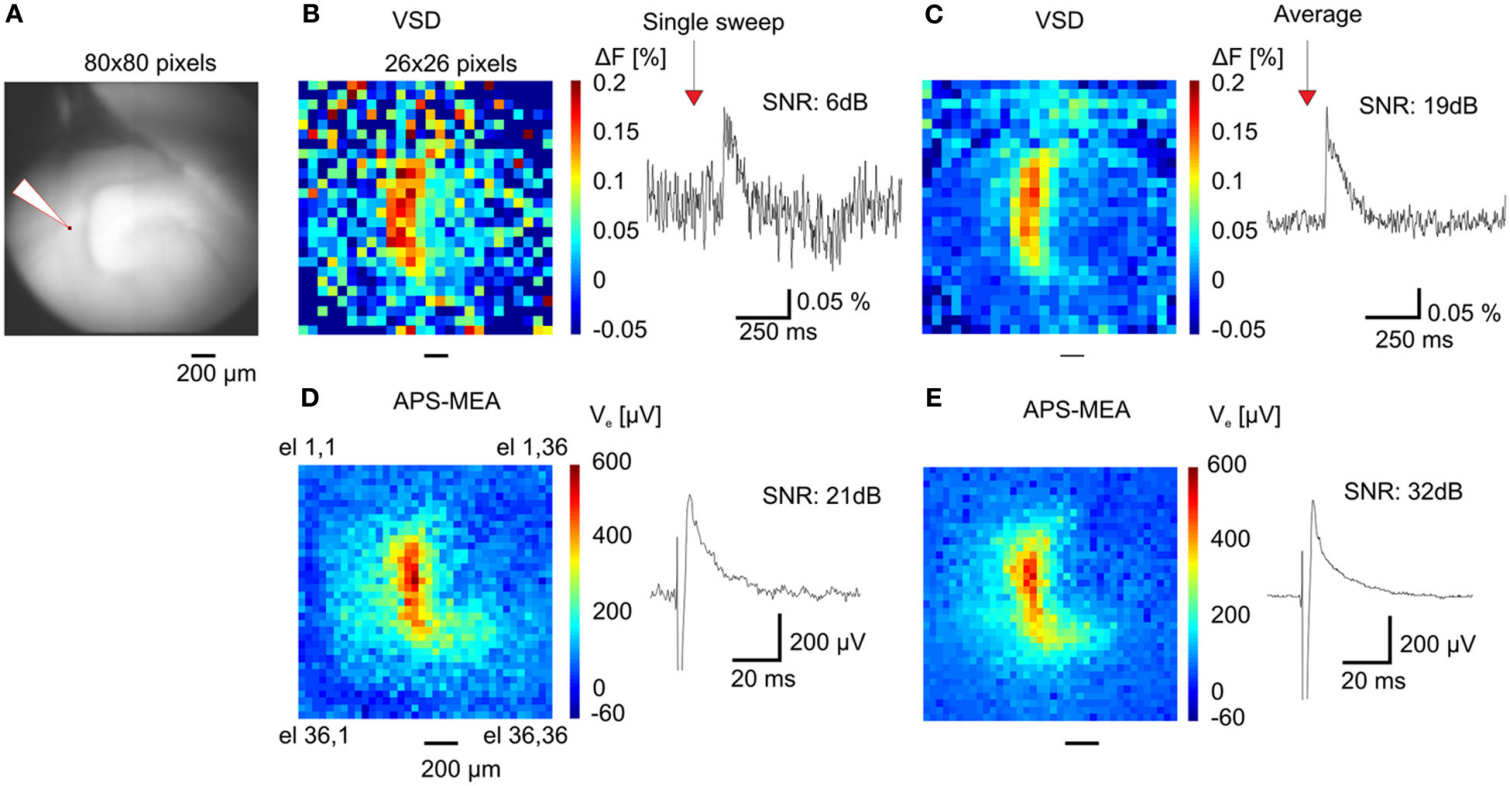
**APS-MEA coupled with VSD recordings. (A)** VSD fluorescence background image of the dentate gyrus. **(B)** Color-coded image of evoked fluorescence changes showing the maximal peak amplitude of the VSD response (left) and a single pixel trace (right). To increase the signal to noise ratio the image is scaled to a 26 × 26 pixel array by averaging nine adjacent pixels. Normalization by a reference frame and bleaching removal was performed on all pixels before averaging. **(C)** Averaged fluorescence image of 20 responses after electrical stimulation (left) and averaged single pixel trace (right). **(D)** Color-coded voltage image showing the peak amplitude of the APS-MEA electrodes after the same electrical stimulation (left) and single electrode voltage trace (right). To compare with VSD recordings, (1) an equivalent field of view is shown by cropping the full array recording, (2) the voltage trace is inverted. **(E)** Color-coded voltage image showing the averaged peak amplitude of 5 responses after electrical stimulation (left) and averaged single electrode voltage trace (right).

When compared with optical recordings such as voltage-sensitive dyes (Grinvald and Hildesheim, 2004) (VSDs), conventional MEAs are superior in signal quality and time resolution, but they fail in terms of spatial resolution because of the small number of electrodes and the large pitch between neighboring channels (usually spaced 200 μm apart). Here, we demonstrate the gain in resolution of our APS-MEA chip by performing simultaneous optical functional recordings with VSDs and electrical APS-MEA recordings from an equivalent field of view of 1.5 × 1.5 mm^2^ (Figure 4A). Upon stimulation of the perforant path, paired recordings of APS-MEA with VSDs show that our method can spatially and temporally resolve single-evoked fEPSPs with a much higher signal-to-noise ratio than single VSD responses (Figures 4B,C vs. Figures 4D,E).

### Electrophysiological imaging of propagating field potentials in epileptic brain slices

**Figure 5:**
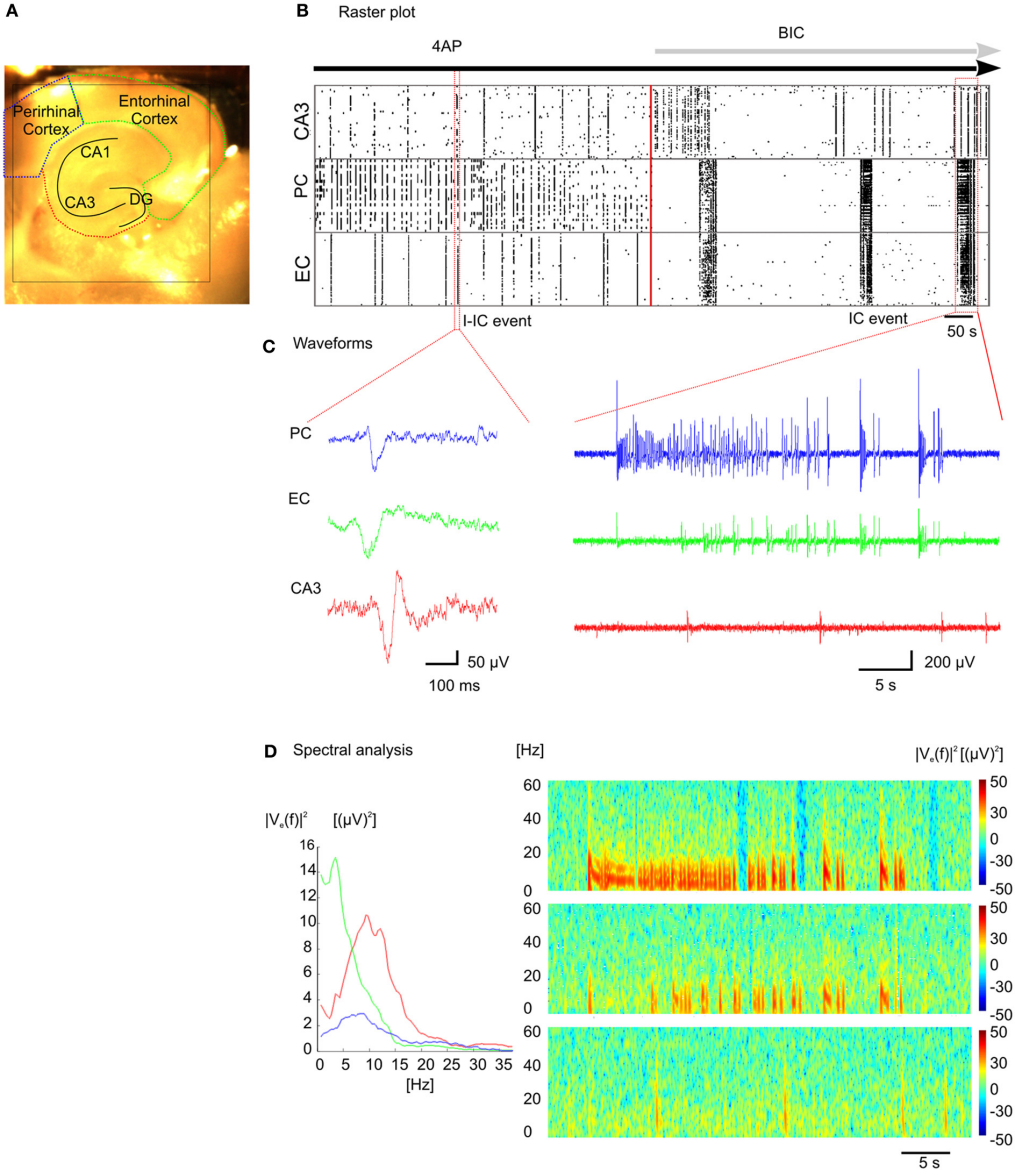
**Recording of epileptic like events. (A)** Cortico-hippocampal slice over the active chip area (black square). **(B)** Raster plot representation of epileptic-like events induced in cortico-hippocampal slice by perfusion with 4-AP ([100 μm]) or 4-AP + BIC ([30 μm]). **(C)** I-IC events in the perirhinal cortex (PC, blue trace), entorhinal cortex (EC, green trace), hippocampus (CA3, red trace), and IC event recorded in the three distinct regions (see Supplementary Video 5). **(D)** Power spectral densities estimated from the I-IC (left) and spectrograms estimated from the IC traces (right).

By taking advantage of the high-density of electrodes integrated over a large active area and the high sampling frequency for each electrode, we have successfully recorded epileptiform activity at high spatio-temporal resolution through the entire slice, encompassing the hippocampal circuit, and part of the connected cortical areas (Figure 5A). We have used 4AP to induce epileptiform activity *in vitro* that resembles Inter-Ictal events (I-IC, Figure 5C, left) recorded in humans with EEG before or after an epileptic seizure (de Curtis and Avanzini, 2001; Avoli and de Curtis, 2011). During analysis, I-IC-like events were identified with a detection algorithm (see “Methods” section) and the time course of the epileptiform activity during the whole experiment is represented in a raster plot (Figure 5B). Noteworthy, distinct anatomical structures show different inter-event latencies. Furthermore, high frequency discharges resembling *in vivo* Ictal (IC) discharges during seizures (Huberfeld et al., 2011) (Figure 5C, right) were recorded after adding bicuculline (BIC). Interestingly, I-ICs were observed in all the anatomical regions of the cortico-hippocampal slice with different shapes (Figure 5C, left) and contents in the spectral frequency band (Figure 5D, left) whereas IC events were observed in perirhinal and entorhinal cortices only. This is also confirmed by the different spectrograms computed from recordings of electrodes located in these distinct regions (Figure 5D, right).

**Figure 6:**
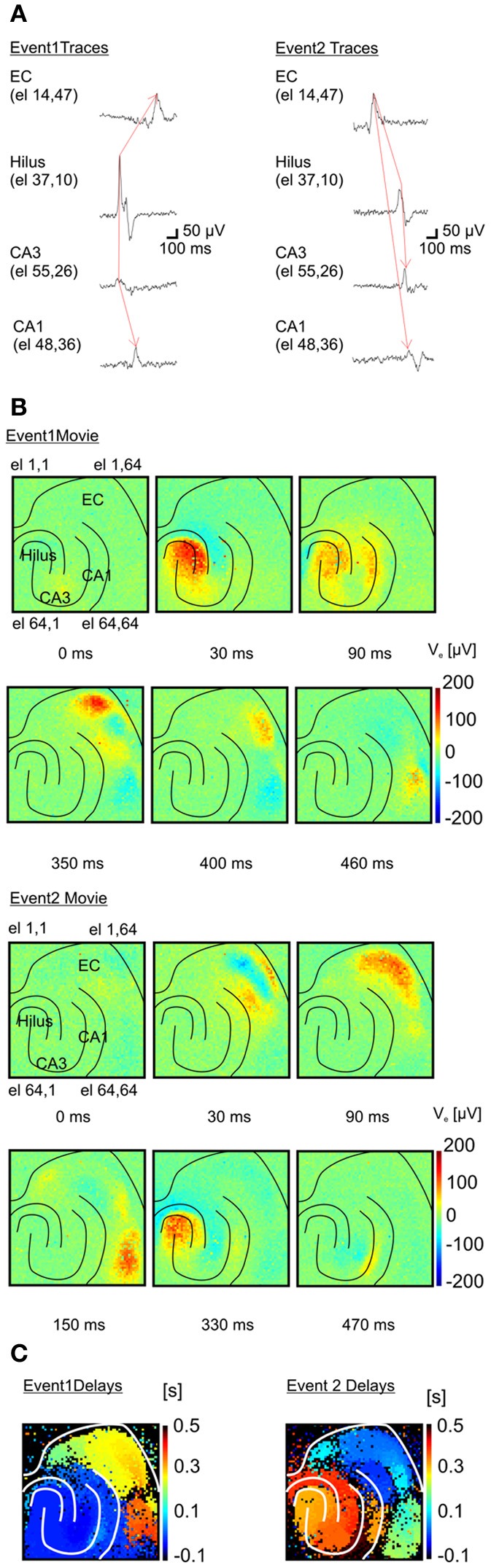
**Functional imaging of an I-IC event propagation. (A)** Two distinct inter-ictal (I-IC) events are represented. Temporal traces corresponding to four different electrodes for each event in different regions: entorhinal cortex (EC), hilus, CA3 and CA1 in the hippocampus. For both events, the red arrow indicates the direction of propagation: event 1 is generated in CA3, propagates to the hilus and afterwards to the EC, whereas event 2 is generated in the EC and propagates to the hilus, CA3 and CA1. **(B)** Time-lapse color-coded voltage maps of extracellular voltages illustrating in detail the different dynamics of propagation for both events. **(C)** Maps of the propagation delays computed for each event with respect to a reference pixel in the region of origin of each event. These maps clearly evidence the two distinct propagations.

Remarkably, the high-resolution of APS-MEAs allows for a detailed description of the dynamic properties of each epileptogenic event (Figure 6). Similar to conventional electrode arrays, the propagation dynamics can be estimated from the time shift of the peak of I-IC activity between a few electrodes located in different brain regions (Boido et al., 2010) (Figure 6A). However, this estimation does not fully describe the dynamics involved in the propagation. Our high-resolution recordings can successfully visualize how activity propagates over the totality of the neural network within the tissue coupled to the electrode array. Extracellular field potentials are represented in 2D with a color-coded scale and image sequences (or movies) are used to depict their spatio-temporal dynamics. Interestingly, different propagation patterns are observed over multiple recorded events. In the example shown in Figure 6B (top row), a first type of I-IC propagation originates in CA3 (*t* = 0 s), propagates to the hilus (*t* = 30 ms), then to CA1 (90 ms) and finally to the entorhinal and perirhinal cortices (*t* = 350 ms, see also Supplementary Video 1). On the other hand, a second type of I-IC propagation (Figure 6B, bottom raw) originates in the entorhinal cortex (*t* = 0 s), propagates to the perirhinal cortex (*t* = 30–150 ms), and finally invades the hippocampus from the DG and CA1 (*t* = 330–470 ms, Figure 6B see also Supplementary Video 2). To summarize these spatio-temporal dynamics, color-coded images of the propagation delays for each event can be computed for all the electrodes in the array by considering a reference electrode located in the site of origin of each event (Figure 6C).

### Characterizing patterns of field potential propagations from multiple recorded events and revealing spatially confined drug-induced functional changes

**Figure 7:**
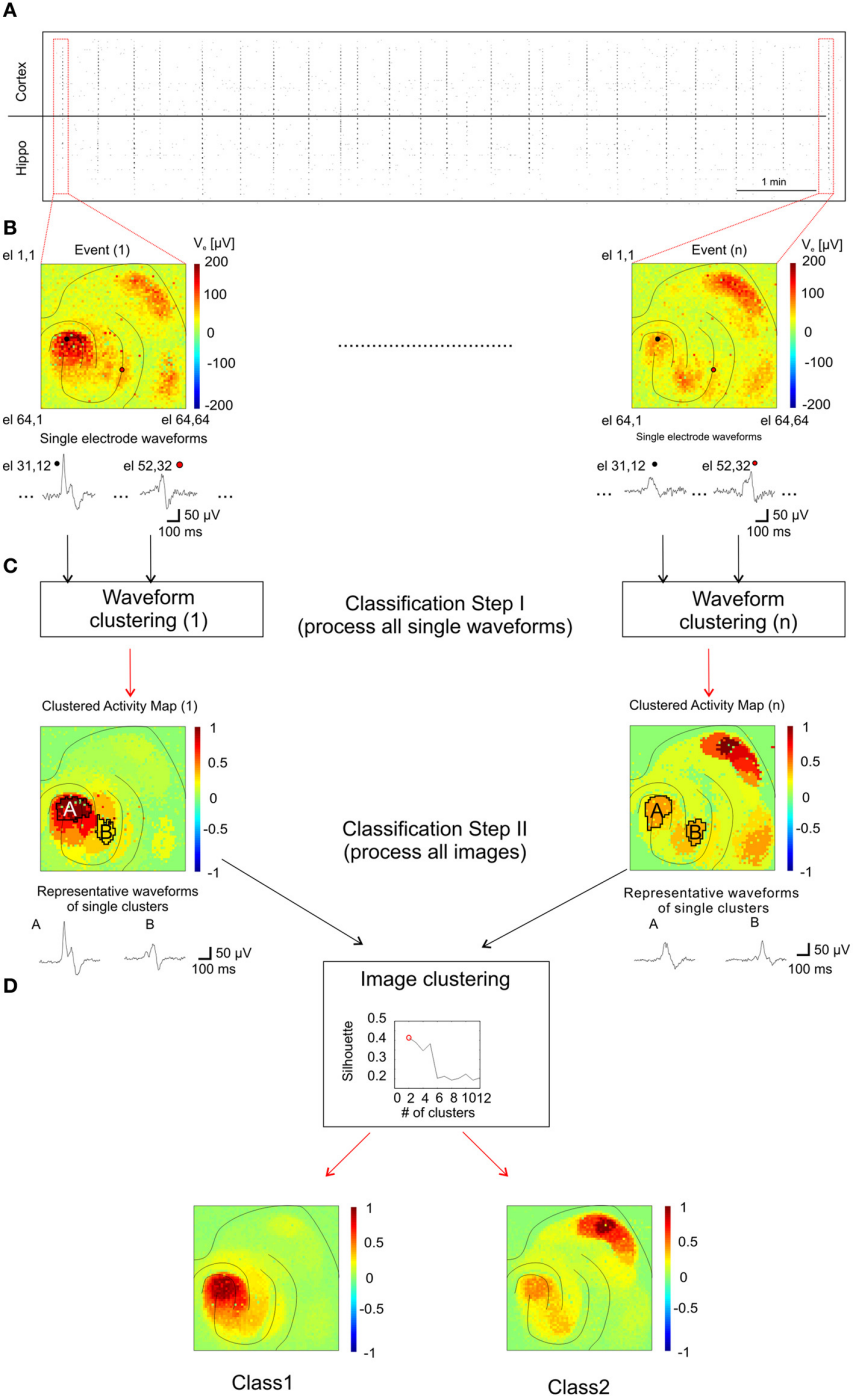
**Analysis method for classification of I-IC patterns. (A)** Raster plot showing the time course of 20 min of recording with dots indicating each detected I-IC for each active electrode. Columns of points therefore indicate single I-IC events recorded in all the active area of the chip. **(B)** Color-map code showing the maximum amplitudes recorded for two different events. The traces are representative of two pixels highlighted with a black and a red circle, respectively. All the electrodes for all the I-IC events were classified with the *kmeans* algorithm. **(C)** Results of the previous classification are shown as spatial map of activation which we referred as CAM. Representative traces are obtained by averaging all recordings from electrodes located in the same region. The CAMs were classified among them on the basis of a clustering criterion which indicates the best number of cluster to fit the data. **(D)** According with the silhouette coefficient, we found out that the optimal number of clusters for all the experiments was 2 (top graph, see Methods section for details). In the bottom panels the averages of all CAM belonging to the two different classes are represented showing the different spatial activation profile of the two classes.

To identify patterns of propagating activities and to correlate them with their corresponding anatomical regions, we developed an analysis method adapted to our large-scale high-resolution recordings (see “Method” section and Figure 7). This was necessary since the spatial extension of I-IC events involve co-activated anatomical regions and the spreading of activity from one region to another follows complex propagation dynamics (Perreault and Avoli, 1992) that cannot be described with a simple trajectory. This propagation complexity is due to the cellular mechanisms of I-IC propagation involving field interactions, gap junction-mediated interactions, and ephaptic propagation (Jefferys, 1995; Frohlich and McCormick, 2010). In our method, classes of I-ICs recorded from each microelectrode were first identified to map regions exhibiting similar waveform shapes, i.e., “Clustered Activity Maps” (CAMs), representing the spatial distribution of each I-IC in the slice (Figure 7C). Successively, to identify distinct I-IC spatio-temporal patterns over *n* recorded events, we classified the *n*-detected CAMs (Figure 7D).

**Figure 8:**
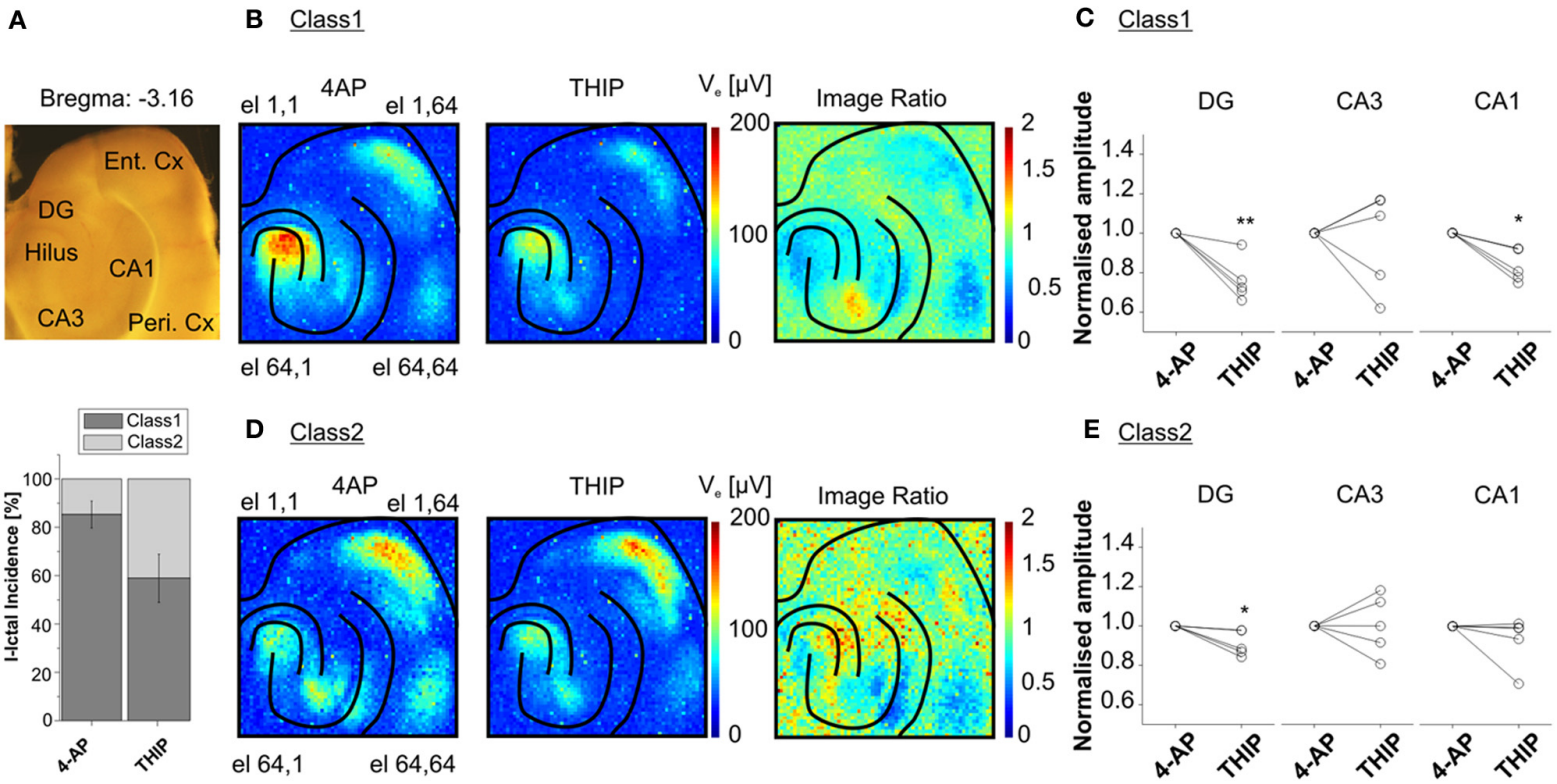
**Classification of I-IC events.** Classification of I-ICs from five experiments reveals that two main classes of I-IC can be identified based on the spatial dissection of the I-ICs (recordings of 20 min. per phase, ~30 total events per phase). Class 1 clusters events originating in CA3 and propagating to other regions of the hippocampus and to the EC, whereas Class 2 clusters events originating in the EC and propagating to the hippocampus. **(A)** On the top, view of the cortico-hippocampal slice over the chip; on the bottom, incidence of the two detected classes over the five different experiments upon treatment with 4AP and THIP. **(B, D)** Average of the maximal amplitude of I-ICs of Class 1 **(B)** and Class 2 **(D)** under 4-AP and THIP ([1μm]). The ratio between THIP and 4AP images shows the spatially circumscribed modulation of THIP on I-IC amplitudes. **(C, E)** Quantification of THIP-induced changes in amplitude of the I-IC events of Class 1 **(C)** and Class 2 **(E)** for electrodes located in DG, CA3, and CA1. The results show that the effect of the compound was statistically significant in DG (24.08 ± 4.84, % of reduction) and CA1 (16.61 ± 3.64, % of reduction) for Class 1 and in DG only (8.94 ± 2.83, % of reduction) for Class 2. ^*^ *p* < 0.05; ^**^ *p* < 0.01; paired Student's *t*-test, *n* = 5 slices from 4 different mice.

Interestingly, our recordings from slices harvested from the same area (from −3.96 to −3.16 mm from Bregma) in several animals showed two major classes of I-ICs, corresponding to distinct propagation patterns in the cortico-hippocampal circuit (Figure 8A). The first class of propagations (Class 1) refers to I-IC events that are generated in CA3 and propagate to the entorhinal and perirhinal cortices (Figure 8B), whereas the second class (Class 2) refers to I-IC events originating in the entorhinal cortex and propagating to the hippocampus and perirhinal cortex (Figure 8D).

High-resolution recordings combined with this analysis method could be used to identify and quantify drug-induced functional changes affecting specific neuronal populations. To demonstrate this unique feature, we performed experiments in the presence of 4,5,6,7-tetrahydroisoxazolo(5,4-c)pyridin-3-ol (THIP), an agonist of the δ-subunit-containing GABA_A_ receptors. These receptors are known to be localized extrasynaptically, and more particularly on DG granule neurons and in CA1 (Scimemi et al., 2005). Therefore, we expected to be able to selectivity induce functional changes in these areas. The activation of these receptors leads to a shift in the membrane potential to more hyperpolarized values (Glykys et al., 2008) and this effect, known as tonic inhibition, represents a protective mechanism against hyperexcitability in the hippocampal network (Semyanov et al., 2003). Interestingly, we found that THIP reduced the amplitude of Class 1 I-ICs in the DG and CA1 regions (i.e., the regions where there is higher expression of GABAA δ-subunit-containing receptors), but not in the rest of the hippocampus (Figure 8C). Moreover, THIP reduced the amplitude of Class 2 I-IC events in DG, but not in CA1 (Figure 8E). These results suggest that the extrasynaptic receptors are selectively recruited in the different circuits involved in the event propagation.

## Discussion

The results presented in this study demonstrate that our 4096-electrode array recording system can literally image brain slices through extracellular field potentials visualized in a color-coded scale, just like traditionally done so far for cellular imaging. As opposed to other systems relying on currently available microelectronic MEA technology, our system was designed to provide near-cellular resolution extracellular field potentials and spiking activity in large-scale networks, allowing us to dissect global network activity with unprecedented detail.

**Figure 9:**
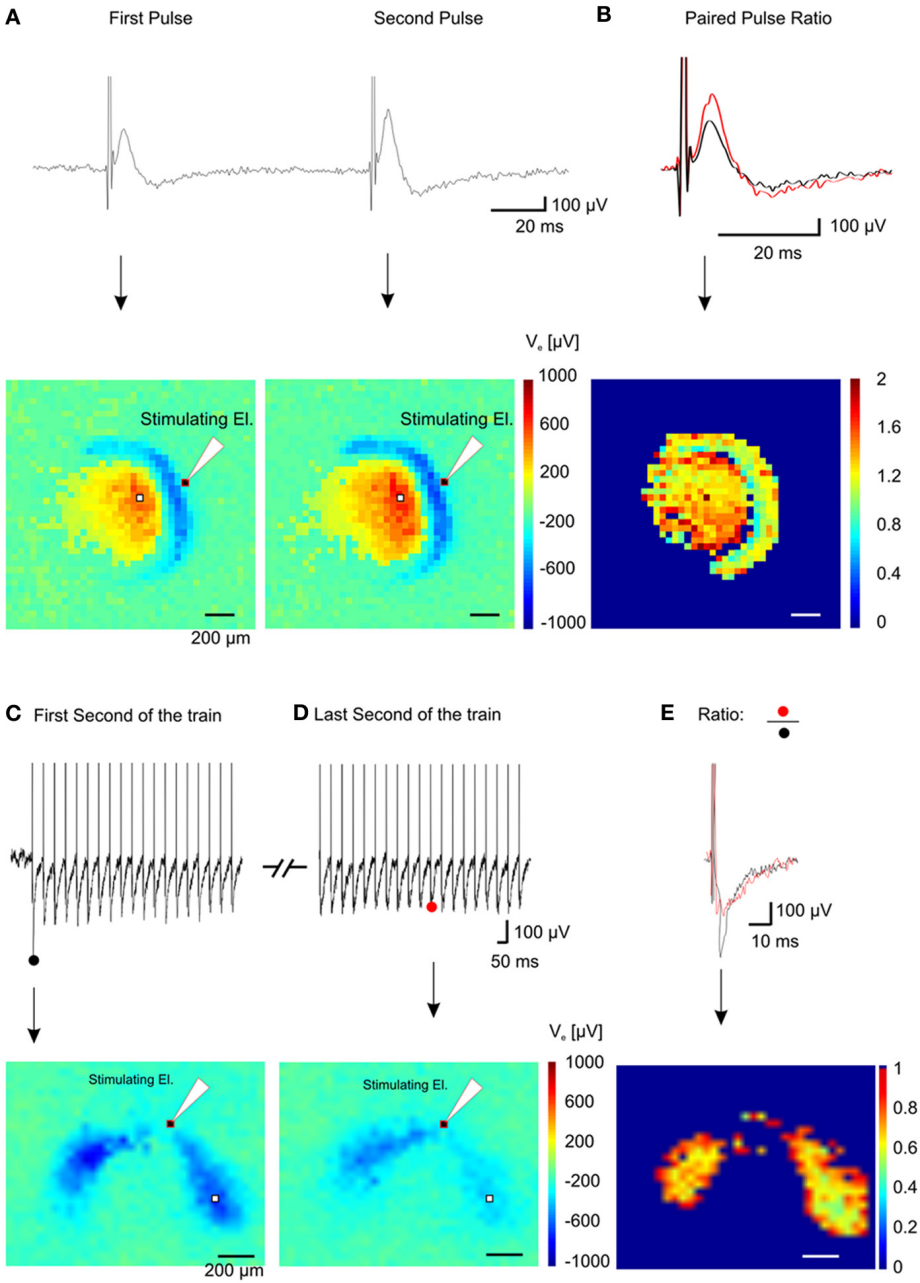
**Spatial short-term plasticity in the dentate gyrus. (A)** Two fEPSPs recorded from a single electrode (white pixel) after a paired stimulation of the lateral perforant path (Δ t = 100 ms) (top trace) and the relative color-coded map of maximum values for the pixel array showing activation of the dentate gyrus and hilus (bottom pictures). **(B)** Superimposition of the first and the second fEPSP shown in **(A)** (top trace) and color-coded spatial distribution of the ratio values recorded in the array (bottom picture). **(C)** Electrical response from one electrode (white pixel in the bottom image) during the first second of stimulation (top trace) of a 10 s train @ 20 Hz and corresponding color-coded minimum peak amplitude (sink in the dendritic layer) of the signal (bottom trace). **(D)** Electrical response from the same electrode during the last second of stimulation (top trace) of the train and corresponding color-coded minimum peak amplitude of the signal (bottom trace). **(E)** Superimposition of the first (black trace) and the last (red trace) fEPSP of the train and color-coded ratio between images in **(D)** and **(E)**, (bottom image).

In order to validate the performance of our system, we have recorded network activity from brain slices encompassing the hippocampus perirhinal and entorhinal cortices. These recordings were performed in combination with patch-clamp and field electrodes as well as with voltage-sensitive-dye imaging. It has to be highlighted that even though we performed experiments on brain slices up to 90 min, we experienced to be able to maintain the viability of the slices for at least 3 or 4 h. In addition to the demonstration of the superior quality of the recordings achieved with our system, we have shown that this MEA system can be combined with other electrophysiological methods. This combined approach, providing complementary high-quality large-scale network and single cell recordings provides unique and novel opportunities to study how LFPs are affected by transmembrane currents of local neurons and vice versa, to understand how these transmembrane currents are involved in shaping the LFP (Buzsaki et al., 2012). In addition, our system allows investigating connectivity and plasticity of neuronal ensembles in great detail, revealing the complex interactions between excitatory and inhibitory network drives. Such potential is illustrated with the use of basic short-term plasticity protocols (Figure 9 and Supplementary Video 3) such as paired and tetanic stimulation.

**Figure 10:**
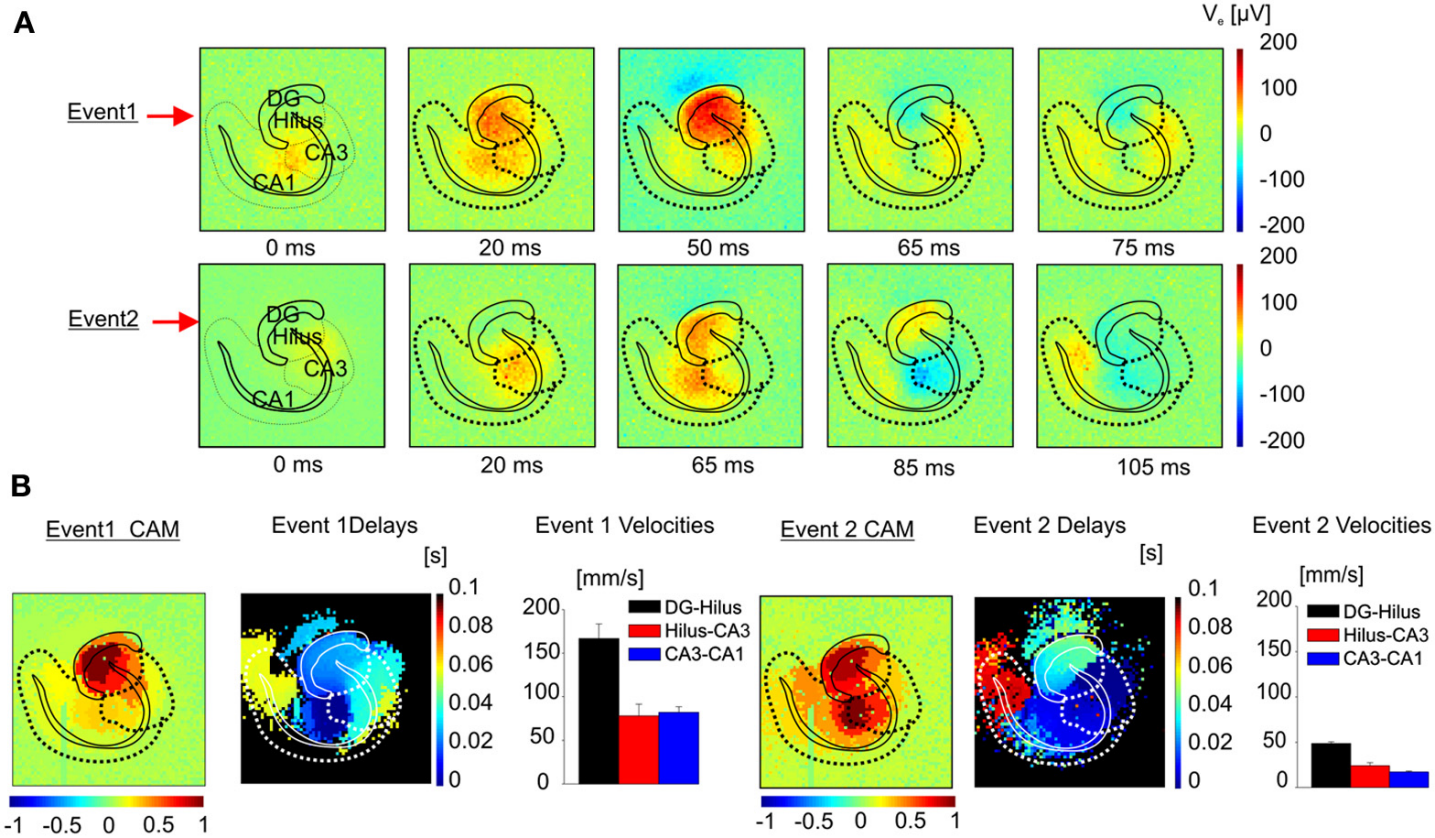
**Classification algorithm applied to I-ICs in the hippocampus. (A)** Color-coded map of extracellular voltage showing the propagation of two different I-IC events. **(B)** For the two events in **(A)** the CAM, the maps of the delays and speeds of propagation e.g., from dentate gyrus to hilus (black bars), from hilus to CA3 (red bars), from CA3 to CA1 (blue bars) are represented. (Event 1 speed: DG-Hilus = 166.6 ± 16.6 mm/s; Hilus-CA3 = 77.8 ± 13.5 mm/s; CA3-CA1 = 81.9 ± 6.6 mm/s. Event 2 speed: DG-Hilus = 48.5 ± 1.1 mm/s; Hilus-CA3 = 24.0 ± 3.5 mm/s; CA3-CA1 = 17.0 ± 1.1 mm/s).

Our electrode array has also been used for imaging pharmacologically induced seizures in brain slices and for characterizing spatio-temporal patterns over multiple recorded events. The ability to image fast propagations involved in epileptogenic events opens new perspectives to clarify how different synaptic and non-synaptic mechanisms are involved in the synchronization of distinct neuronal populations (Jefferys et al., 2012) and in the propagation of the IC and I-IC events. Indeed, the I-ICs propagation dynamics clearly differs from the dynamics of purely synaptic events, such as electrically evoked LFP or spontaneous action potentials (APs). This important aspect can be better appreciated by comparing functional images of I-IC events (Figure 6 and Supplementary Video 1, 2) with electrically evoked LFPs (Figure 1 and Supplementary Video 3) and with APs (Figures 2A,B and Supplementary Video 4). Interestingly, functional imaging of I-IC dynamics shows simultaneous activation of large areas due to the local synchronous firing of many neurons that contribute in shaping the extracellular field potential (McCormick and Contreras, 2001) and establishing a distinct spatial distribution. Moreover, our recordings reveal that areas recruited during spontaneous field potential propagation involve adjacent functionally connected anatomical regions, but the latencies in signal propagation from one region to another not always directly correspond to specific synaptic connections. On the contrary, LFPs evoked in the dendritic layer, granular cell layer, and polymorphic layer through extracellular stimulation of the perforant pathway appear to involve shorter activation latencies, involving synaptic-mediated propagation. In the case of APs recorded in the hippocampus at the level of the cell bodies and axons, we observed propagations compatible with synaptic transmission from CA2 to CA3, CA1, and DG during bursting activity (Figures 2A–D and Supplementary Video 4). Thus, given the complex connectivity involved in I-IC event propagations, to identify patterns of I-IC propagations from high-resolution recordings we have developed a novel analytical approach consisting of pan-array mapping of the spatial distribution of extracellular field potentials having a similar shape in distinct anatomical regions and propagating through the circuit with the same sequential activation of local regions. This analysis approach of network dynamics is possible only when applied to high spatio-temporal resolution recordings such as those provided by the APS-MEA. Indeed, since the local spatial distribution of I-ICs varies depending on the propagation pattern that locally activate a region, we can identify dissimilar spatial distributions of local activity patterns in order to determine how different areas of the global network are affected by different propagation patterns. This is an important aspect of our analysis since it unmasks the existence of the relationship between local activations and propagation patterns that has been previously overlooked. Over multiple I-IC recorded events, we have identified two distinct classes of I-ICs corresponding to two distinct propagation patterns originating either from the hippocampus or from the entorhinal cortex. This was achieved by recording from brain slices harvested from several mice and cut on the same plane to ensure reproducibility. However, when slices were harvested from a different height (e.g., at −4.44 mm from Bregma; Figure 10), we observed different classes with distinct trigger focal points and propagation velocities, most probably due to the diverse circuits present in these slices from different anatomical locations.

Finally, our analysis method can effectively reveal and quantify spatially confined functional changes induced by a drug. To validate this capability, we used THIP to target δ-subunit containing GABA_A_ receptors in the cortico-hippocampal circuit. Interestingly, the spatial specificity of the effect of the compound could be separately evaluated for different classes of I-IC propagations. Distinct THIP effects for different I-IC classes were observed. For the first class, we found that the THIP effect was remarkably confined to the molecular layer of the DG and to the stratum pyramidal and stratum oriens of CA1. For the second class the compound was found effective only in the molecular layer of the DG, but not elsewhere. These differences might be explained on one hand by the different number of THIP-targeted receptors expressed in different regions of the brain slice, and on the other hand, by the different propagation patterns involving diverse local neuronal connectivity. In particular, we observed that I-IC events of Class 1 originating in CA3 propagate synaptically to the DG with a delay lower than 30 ms (see Figures 6B,C top raw), whereas I-IC events of Class2 originating in the EC propagate in the DG with a delay greater than 300 ms (see Figures 6B,C bottom raw). The latter is unlikely to be synaptically mediated and, given the large propagation delay, most probably involves non-synaptic processes such as diffusion of extracellular potassium (Lian et al., 2001; Avoli et al., 2002). Overall, these results show that our electrode array and analysis approaches provide important information to spatially evaluate the effects of a drug in specific regions, or even dynamically in the context of the spreading activity. As far as both dynamic spreading activity and mechanism(s) of propagation are concerned, parameters such as speed of propagation or area of activation will vary from event to event and will respond differently to pharmacological treatments, depending on the class they belong to.

In summary, the novel recording and analytical approaches that we have presented here to characterize epileptic events could, in principle, be applied to investigate neuronal network dynamics in many other systems. Furthermore, our method can be uniquely combined with other experimental approaches such as optogenetics or 3D fluorescence microscopy for detailed studies spanning the spatial scale from single neurons up to the entire circuit level (Helmchen and Denk, 2005).

## Methods

### Animals and brain slices preparation

All experiments were performed on C57BL/6J mice of either sex aged 3 weeks to 6 months (Charles River Laboratories International, Wilmington, MA, USA). All experiments were carried out in accordance with the guidelines established by the European Community Council (Directive 2010/63/EU of September 22nd, 2010) and experimental protocols were approved by the Italian Ministry of Health. Animals were anaesthetized with isofluoran prior to decapitation. Transverse hippocampal slices (400 μm thick) were cut using a Microm HM 650 V microtome equipped with a Microm CU 65 cooling unit (Thermo Fisher Scientific, Waltham, MA). Slices were cut at 2°C in a high-sucrose protective solution containing (in mM): 87 NaCl, 25 NaHCO_3_, 2.5 KCl, 0.5 CaCl_2_, 7 MgCl_2_, 25 glucose, 75 sucrose, and saturated with 95% O_2_ and 5% CO_2_. Slices were incubated for 30–45 min at 35°C and for at least another hour at room temperature in recording solution prior to being used for recordings (see in “*Patch-Clamp and Single Field Recording”* session).

### High-density active-pixel sensor recordings

The APS-MEA system was extensively described in previous papers (Imfeld et al., 2008). Briefly, it consists of a CMOS-based CCD monolithic chip modified such that pixels are designed to sense electrical voltage variations induced by electrogenic tissues. The chip integrates amplification and analog multiplexing circuits designed to provide simultaneous extracellular recordings from 4096 electrodes at a sampling rate of 7.7 kHz per channel. Each square pixel measures 21 × 21 μm, and the array is integrated with an electrode pitch (center-to-center) of 42 μm. Pixels are arranged in a 64 × 64 array configuration, yielding an active area of 7.22 mm^2^ with a pixel density of 567 pixel/mm^2^. The three on-chip amplification stages provide a global gain of 60 dB, with a 0.1–5 kHz band-pass filter. This bandwidth is adapted to record slow LFP signals as well as fast APs. Acquisition is controlled by the software BrainWave (*3Brain Gmbh, Switzerland*).

### Patch-clamp and single field recordings

Both field and whole-cell patch-clamp recordings were performed with a Multiclamp 700B/Digidata1440A system (*Molecular Devices, Sunnyvale, CA, USA*) on an upright Olympus BX51WI microscope (*Olympus, Japan*) equipped with Nomarski optics and reflected light. Slices were bathed in artificial cerebrospinal fluid (ACSF) containing (in mM): 125 NaCl, 25 NaHCO_3_, 25 glucose, 2.5 KCl, 1.25 NaH_2_PO_4_, 2 CaCl_2_, and 1 MgCl_2_ (bubbled with 95% O_2_–5% CO_2_). The solution was perfuse at a rate of 2.5 ml/min. For whole-cell patch-clamp recordings we used borosilicate glass electrodes (*Kimble Chase, Vineland, NJ, USA*). The patch electrode resistance was between 4 and 6 MΩ. Recordings were done in selected granule neurons from the granule layer of the DG in the presence of 4-aminopyridine (4-AP, [100 μm]) and at a holding potential (V_h_) of −80 mV. The intracellular solution contained (in mM): 126 K-gluconate, 4 NaCl, 1 MgSO_4_, 0.02 CaCl_2_, 0.1 BAPTA, 15 Glucose, 5 HEPES, 3 ATP, and 0.1 GTP in which the pH was adjusted to 7.3 with KOH and the osmolarity to 290 mosmol·l^−1^ with sucrose. For patch-clamp recordings the current traces were sampled at 50 kHz, filtered at 10 kHz, and stored for off-line analysis. Field recordings were performed in various areas of the hippocampus and DG. For field recordings, we used borosilicate glass electrodes (*Kimble Chase, Vineland, NJ*) with an internal resistance of 1–2 MΩ and filled with extracellular recording solution (see composition above). The acquisition was also performed with the Multiclamp 700B/Digidata1440A system after a pre-amplification with a home-made pre-amplifier of 40 dB. The total gain was set to 60 dB. For stimulation experiments of the DG, we used a monopolar stimulation electrode placed on the perforant path connected to an external stimulator (*A-M Systems, Sequim, WA*). Unequivocal evoked responses, well separated from the stimulation artifact, were obtained using stimulation pulses of 200–400 μA lasting 30 μs.

### Voltage-sensitive dye recordings

The VSD RH-795 (4 μg/ml) was used to label cell membranes. Hippocampal slices were incubated in a chamber containing 2 ml of ACSF and RH-795 for 15 min. After labeling, slices were transferred to ACSF solution for 10 min to wash out residual VSDs. A high-resolution (80 × 80 pixels) CCD camera (*RedShirtImaging, Tokyo, Japan*) was used to sample membrane potential depolarization at a frame rate of 2 kHz. Extracellular stimulation was applied as described in the *Patch-Clamp and Single Field Recording* section. Cells were imaged with wide field epifluorescence using a 10× water immersion objective. Baseline correction for bleaching, spatial averaging, and trace averages were computed off-line with custom-written Matlab scripts.

### Recording of spontaneous and electrically evoked activity

Acute cortico-hippocampal slices were recorded for 20 min per session (once activity had stabilized for at least 30 min). Spontaneous LFPs were elicited by bath application of convulsant agent 4-AP ([100 μm]) (Avoli, 1990). Epileptiform discharges were also modulated by the GABA_*A*_ channel blocker BIC ([30 μm]) or THIP, ([1 μ M]), an agonist of the δ-subunit-containing GABA_A_ receptors.

### Data analysis

All the analysis algorithms used in this study were developed as Matlab scripts (*MathWorks*, *http://www.mathworks.it/*) with the exception of the field potential event detection that has been implemented in C# language under Visual Studio (*http://www.microsoft.com/visualstudio/en-gb*). The computational facility for the most intensive computations (e.g., k-means classification of signal shape among 4096 electrodes) was provided by the parallel cluster CASPUR (*Consorzio Interuniversitario per le Applicazioni di Supercalcolo per Università e Ricerca* - *http://www.caspur.it/en/*, project STD11-499).

### Field potential event detection

To evaluate evoked field responses and compare paired-pulse responses (Figures 1, 3, and 9), we calculated the peak (absolute value) of evoked extracellular signals. Raster plots (Figures 5 **and** 7) show the events detected by the previously described Precision Timing Spike Detection (PTSD) algorithm (Maccione et al., 2009). The algorithm, originally tailored to detect fast spiking activity generated by a few neuronal units, has been adapted to detect slower field potential events. To this purpose, the threshold was set to 5 times the standard deviation of the noise, while the refractory period and the peak lifetime period were set to 50 ms and 40 ms respectively.

### Estimation of time delays and propagation velocities of Inter-Ictal events

Color-coded maps of the time delays occurring during the propagation of an I-IC event were computed by calculating the cross-covariance of one electrode (taken as reference) selected in the focus of activity (i.e., the area where the event originates) with respect to any other electrode on the array. The time delay of the cross-covariance peak was used to reconstruct the color-coded maps of the time delays. Cross-covariance values falling below a certain threshold value (e.g., 0.3) presumably did not represent propagation and were discarded by setting the color to “black” (data not shown). Velocities were calculated from time delays and distances of 4 electrodes selected as: two electrodes representative of the starting and ending regions, and two electrodes involved in the propagation to better approximate the non-linearity of the spatial and temporal distribution of the event. The spatial trajectory covered by the event was estimated as the sum of the four Euclidean distances between the consecutive selected electrodes. Then, the distance was divided by the time delay of the starting and ending electrode as calculated for the color-coded maps. Propagation velocities were estimated by averaging 7 events showing the same trajectory of propagation.

### Classification of the spatial and temporal distributions of Inter-Ictal events

I-IC event classification presented in Figures 8B,D was calculated by following the procedure steps illustrated in Figure 7. I-IC events were manually detected by identifying synchronous events (Figure 7A) in the raster plot representation. The activity of a single I-IC is visualized in a false color map, where the most active electrodes are red colored. Figure 7B shows two examples of different I-ICs where electrode channels (e.g., 31,12 and 52,32) show different signal shapes. Each event was cut-off in a separate subset of 1 s time windows. For all 4096 electrodes, the signals were denoised using a band pass filter (1–100 Hz), corresponding to the I-IC frequency band content (see Figure 5D). All further steps were made by joining the dataset obtained under the two treatments (4-AP and 4-AP + THIP).

For each event, we first classified the signal waveforms among the electrodes in order to cluster events with similar waveforms (Figure 7C). The classification process was based on k-means algorithm (Xu and Wunsch, 2005). We initially set the maximum number of classes (i.e., in how many clusters signals had to be separated) to 20. Clusters populated by less than 5 events (on average 3–4 clusters) and/or signals with amplitudes less than 20 μV were discarded. Then, all clusters were associated to a representative waveform (i.e., the averaged waveform of all electrode waveforms in the same cluster, as shown in Figure 7C) and displayed in color code maps showing the peak-to-peak amplitude of the their representative waveform normalized to the highest peak-to-peak value between all representative waveforms (two illustrative clusters and representative waveforms named A and B, are indicated in Figure 7C). This classification procedure yielded a color-coded CAM for each I-IC event (Figure 7C, Classification Step I) that represents the spatio-temporal distribution of signals based on their waveform similarity. Importantly, even if k-means is a “blind” algorithm (i.e., it does not consider the precise spatial arrangement of the electrodes), the signals belonging to a cluster are almost all contiguous and cluster distributions generally overlap well with the anatomical organization of the slice. In a second classification step, CAMs were computed under different experimental conditions (i.e., 4AP and 4AP + THIP), collected and classified (Figure 7D) following the same principle previously described (Gandolfo et al., 2010). For this second classification step, we varied the number of clusters between 2 and 15 and found that the optimal number of clusters for all the experiments was 2, according to the silhouette coefficient introduced by Kaufman and Rousseeuw to test clustering efficiency (Kaufman and Rousseeuw, 1990).

### Estimation of the functional changes in distinct hippocampal areas

To estimate functional changes occurring after chemical manipulation of *n* = 5 experiments, we identified three distinct areas of the hippocampus, i.e., DG, CA3, and CA1 including only the *stratum pyramidale* and the *stratum oriens*. For each event, we averaged signals among electrodes belonging to the identified area (Figures 8B,D). As an example, histograms in Figures 8C,E show the ratio between the averaged signal peak for events in 4-AP and for the events in 4-AP + THIP.

### Statistical analysis

Statistical analysis was performed using Origin 8.0 (*OriginLab Corporation, Northampton, MA*). We used paired Student's *t*-test to assess the effect of THIP in various hippocampal regions. A *p*-value lower than 0.05 was considered statistically significant (^*^p < 0.05, ^**^p < 0.01). Data throughout the study are shown as mean ± SEM, where the means represent the average of the mean values calculated from each individual slice.

## Author contributions

E. Ferrea, L. Medrihan, A. Maccione, L. Berdondini designed and carried out the experiments, developed the algorithms and analyzed the data. D. Ghezzi contributed with VSD experiments and analysis. T. Nieus supported the analysis on the computing cluster and contributed in writing the method section. P. Baldelli, F. Benfenati gave conceptual advice and reviewed the manuscript. E. Ferrea, L. Medrihan, A. Maccione, and L. Berdondini prepared the manuscript.

### Conflict of interest statement

The authors declare that the research was conducted in the absence of any commercial or financial relationships that could be construed as a potential conflict of interest.

## Supplementary Material

**Video S1 | Movie of a first type of recorded I-IC propagation originating in CA3 (*t* = 0 s), propagating to the hilus (*t* = 30 ms), then to CA1 (90 ms) and finally to the entorhinal and perirhinal cortices (*t* = 350 ms).** On the left, raw traces of three selected electrodes located in the Hilus, Ent-CX, CA1. On the right, color-coded extracellular activity.

**Video S2 | Movie of a second type of recorded I-IC propagation originating in the entorhinal cortex (*t* = 0 s), propagating to the perirhinal cortex (*t* = 30–150 ms) and finally invading the hippocampus from the DG and CA1 (*t* = 330–470 ms).** On the left, raw traces of three selected electrodes located in the Hilus, Ent-CX, CA1. On the right, color-coded extracellular activity.

**Video S3 | Movie of evoked electrical response induced by paired pulse stimulation (inter-pulse interval Δ*t* = 100 ms) in the dentate gyrus.** On the left, raw trace of one electrode located in the hilus. On the right, color-coded extracellular activity.

**Video S4 | Movie of recorded spontaneous spiking activity during a burst (duration 150 ms).** On the left, raw trace of one electrode located in CA2. On the right, color-coded extracellular activity.

**Video S5 | Movie of IC propagation originating in the perirhinal cortex and propagating in the entorhinal cortex and sometimes invading the hippocampus.** The recorded duration is of 30 s.
